# Successful Reversal of Severe Tachycardia-Induced Cardiomyopathy with Cardiogenic Shock by Urgent Rhythm or Rate Control: Only Rhythm and Rate Matter

**DOI:** 10.3390/jcm10194504

**Published:** 2021-09-29

**Authors:** Kim Volle, Clément Delmas, Anne Rollin, Quentin Voglimacci-Stephanopoli, Pierre Mondoly, Eve Cariou, Franck Mandel, Hubert Delasnerie, Maxime Beneyto, Michel Galinier, Yoan Lavie-Badie, Didier Carrié, Jerôme Roncalli, Olivier Lairez, Pauline Fournier, Caroline Biendel, Philippe Maury

**Affiliations:** 1 Department of Cardiology, University Hospital Rangueil, 31400 Toulouse, France; volle.k@chu-toulouse.fr (K.V.); delmas.c@chu-toulouse.fr (C.D.); rollin.a@chu-toulouse.fr (A.R.); voglimacci.q@chu-toulouse.fr (Q.V.-S.); mondoly.p@chu-toulouse.fr (P.M.); cariou.e@chu-toulouse.fr (E.C.); mandel.f@chu-toulouse.fr (F.M.); hubertdelasnerie@gmail.com (H.D.); beneyto.maxime@gmail.com (M.B.); galinier.m@chu-toulouse.fr (M.G.); lavie-badie.y@chu-toulouse.fr (Y.L.-B.); carrie.d@chu-toulouse.fr (D.C.); roncalli.j@chu-toulouse.fr (J.R.); lairez.o@chu-toulouse.fr (O.L.); fournier.p@chu-toulouse.fr (P.F.); biendel.c@chu-toulouse.fr (C.B.); 2 I2MC, INSERM UMR 1297, University of Toulouse III, 31432 Toulouse, France

**Keywords:** cardiogenic shock, tachycardia-induced cardiomyopathy, catheter ablation, heart failure, atrial fibrillation, atrial flutter

## Abstract

Background and objectives Severe forms of Tachycardia-induced cardiomyopathy (TIC) with cardiogenic shock are not well described so far, and efficiency of catheter ablation in this setting is unknown. Methods We retrospectively included consecutive patients admitted to the Intensive Cardiac Care Unit for acute heart failure with cardiogenic shock associated with atrial arrhythmia and managed by ablation. Result Fourteen patients were included, each with cardiogenic shock and two needing the use of extracorporeal membrane oxygenation. Successful ablation was performed in the acute setting or over the following weeks. Two patients experienced relapses of arrhythmias and were treated by new ablation procedures. At 7.5 ± 5 months follow-up, all patient were alive with stable sinus rhythm. The left ventricular Ejection Fraction dramatically improved (21 vs. 54%, *p* = 0.001) as well as the end-diastolic left ventricular diameter (61 vs. 51 mm, *p* = 0.01) and NYHA class (class IV in all vs. median 1, *p* = 0.002). Conclusion Restoration and maintenance of sinus rhythm in severe TIC with cardiogenic shock and atrial arrhythmias lead to a major increase or normalization of LVEF, reduction of ventricular dimensions, and improvement in functional status. Ablation is efficient in long-term maintenance of sinus rhythm and may be proposed early in refractory cases.

## 1. Introduction

Heart failure affects 1–2% of subjects in developed countries, concerning more than 10% of people over the age of 70 [1], representing a leading cause of morbidity and mortality. Identifying a potentially reversible cause of heart failure and improving and eventually normalizing left ventricular systolic function is therefore of major interest.

Tachycardia-induced cardiomyopathy (TIC) is a well-known reversible cause of heart failure. TIC was described as early as 1949 by Philip and Levine, after observing that rapid atrial fibrillation (AF) can induce transient heart failure in patients with otherwise normal hearts [2]. TIC can be defined as a reversible left ventricular systolic alteration secondary to persisting or incessant rapid tachycardia [3,4,5]. The diagnosis is established by demonstrating recovery of LV function after elimination of the arrhythmia or control of the ventricular rate. Any arrhythmia sufficiently fast and prolonged can be responsible for TIC [4], but AF is the leading cause of TIC in adults [4]. Despite its recognition for many years, the impact of TIC is still often underestimated [3] although prevalence seems relevant: it is present in 10% of adults and 38% of children with atrial tachycardia [6,7,8].

AF is present in 10–50% of patients with heart failure, worsening symptoms and impairing left ventricular systolic function due to poor ventricular rate control [4,9]. Many patients with AF and heart failure likely have a component of TIC [4]. Catheter ablation has been repetitively shown to improve left ventricular function and to enhance the outcome of patients with heart failure and AF in major studies [10,11,12].

TIC can lead sometimes to severe alteration of LVEF, and in the most severe forms, to cardiogenic shock and sudden death [13,14,15,16]. However, these severe cases remain marginal and not well described. We conducted an observational study about these “severe” TIC defined by cardiogenic shock and the impact of catheter ablation on LVEF, functional status, and mortality. 

## 2. Methods

We conducted a retrospective observational study at the University Hospital Toulouse France. We included all successive unselected patients admitted to the Intensive Cardiac Care Unit between 2018 and 2020 for severe heart failure and cardiogenic shock expected to be caused by TIC and who had undergone percutaneous radio-frequency (RF) ablation of atrial arrhythmias as a bail-out therapy.

Although no firm criteria exist, TIC could be reasonably suspected in the case of supraventricular tachycardia expected to be of recent occurrence (no previous history of permanent tachycardia and/or no symptoms evocative of permanent arrhythmias before) occurring in patients without other specific causes of cardiogenic shock (no ischemic heart disease, no history of cardiomyopathy, no valvular disease, etc.).

Cardiogenic shock was diagnosed using a combination of three criteria as defined in the FRENSHOCK registry [17]:

(1) low cardiac output (low cardiac index < 2.2 L/min/m^2^ and/or systolic blood pressure < 90 mmHg or vasopressors/inotropes needed to maintain systolic blood pressure > 90 mmHg).
(2) right and/or left ventricular overload defined by congestive clinical features, natriuretic peptide elevation, radiological, echocardiographic, and/or hemodynamical parameters.
(3) organ malperfusion defined by clinical (oliguria, confusion, pale and/or cold extremities, mottled skin) and/or biological features (lactate > 2 mmol/L, metabolic acidosis, renal failure, liver insufficiency).

The presence of clinical signs of heart failure and cardiogenic shock was noted by physical examination on admission, over the hospitalization, and during follow-up.

Clinical data were collected, together with a previous history of heart disease, cardio-vascular risk factors, and comorbidities.

The cardiac rhythm was assessed on ECGs done at admission and during the hospitalization, and the duration of arrhythmia history was sought by interrogation or by checking the patient’s medical records when available.

Left ventricular ejection fraction (LVEF) and end diastolic diameters (EDLVD) had been measured using transthoracic echocardiography (TTE) at admission, over the hospitalization, and at follow-up. Right ventricular function was evaluated using TAPSE, S wave, and systolic pulmonary artery pressure. MRI had been performed in some patients after the acute phase. Data from invasive hemodynamical investigations were retrieved when performed.

Biological monitoring included blood gaz and arterial lactate, prothrombin ratio, NT-pro-BNP, thyroid stimulating hormone (TSH), aspartate aminotransferase (ASAT), alanine aminotransferase (ALAT), factor V Leiden, total bilirubin and creatinine levels, and estimated glomerular filtration rate (eGFR).

The mean Simplified Acute Physiology Score (SAPS) was calculated [18].

Ablation was planned in these patients after failure of acute anti-arrhythmic drug therapy as a last resort step for avoiding potentially unnecessary and aggressive therapy such as urgent transplantation or chronic mechanical circulatory support. Catheter ablation was performed with radio-frequency and according to the current techniques and end-points [19].

Short-term (before discharge) or long-term (after discharge) follow-up was assessed using files and consultations with physicians. Follow-up was censored in terms of survival, relapse of arrhythmia, recurrence of acute heart failure, need for chronic mechanical circulatory support or transplantation.

According to the French ethic and regulatory law, retrospective studies based on the exploitation of usual care data do not need to be submitted to an ethical committee but have to be covered by reference methodology of the French National Commission for Informatics and Liberties (CNIL). After evaluation and validation by the data protection officer and according to the General Data Protection Regulation, this study completing all the criteria, was registered in the register of retrospective studies of the Toulouse University Hospital (number RnIPH 2020-146) and covered by the MR-004 (CNIL number: 2206723 v 0). This study was approved by Toulouse University Hospital, and we confirm that ethic requirements were totally respected in the above report.

### Statistical Analyses

Continuous variables are reported as the median and interquartile ranges (IQR) and were compared with Mann Whitney (or Wilcoxon) nonparametric tests for unpaired (or paired) comparisons. Categorical variables are reported as a number and % and were compared using Fisher’s exact test. Analysis and calculations were performed using the StatView™ program (Abacus Concepts, Inc., Berkeley, CA, USA, 1992–1996, version 5.0). A *p* value < 0.05 was considered statistically significant.

## 3. Results

**Figure 1:**
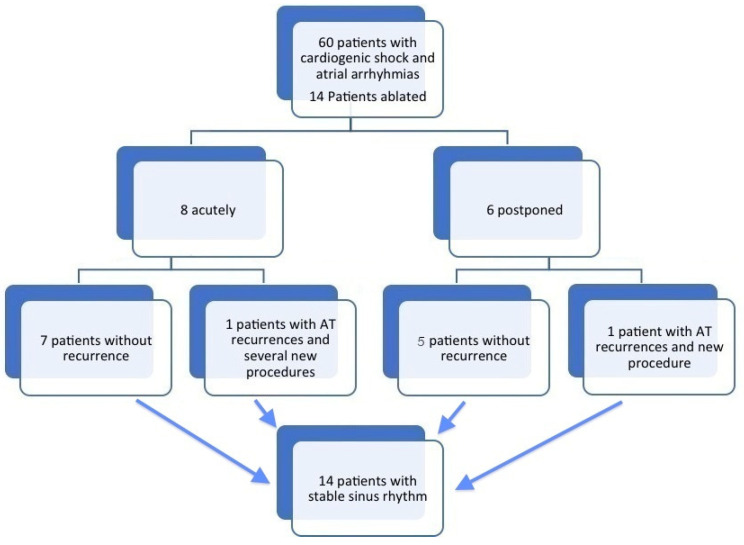
Flow-chart of the included patients.

Fourteen patients were included between 2018 and 2020 (13 men, median 53 years old, IQR 11). During the same period of time, around 400 patients were admitted for cardiogenic shock at our institution, and around 60 were in AFib. Apart from the 14 patients included here, no ablation was performed in any of them at this time (see flow-chart Figure 1).

At admission, all patients presented with supraventricular arrhythmia on ECG: AF (*n* = 9), common atrial flutter (*n* = 3), AF and flutter (*n* = 1), and atrial tachycardia (AT) (*n* = 1). In five cases, there was a previous history of paroxysmal tachycardia, lasting for 1 month, 21 months, 2, 3, and 8 years (two patients underwent AF and flutter ablation 8 years and 21 months before). A TTE had been previously performed in four patients because of arrhythmias or for incidental reasons, showing normal LVEF (55%, IQR 10). There was no other past cardiac history in any patient. No patient presented with previous peripheral arteriopathy, renal failure, stroke or transient ischemic attack.

Comorbitities and cardio-vascular risk factors are listed in Table 1.

One patient was on rivaroxaban for a previous diagnosis of AF, and one patient received a calcium channel blocker for hypertension. The remaining patients were not receiving any cardiologic drug therapy.

Blood pressure at admission was 102 mm Hg (IQR 25) for systolic and 73 mm Hg (IQR 24) for diastolic arterial pressure (available for five cases only while on catecholamine support). The mean heart rate was 125 bpm (IQR 49, from 56 to 170), and all patients were in NYHA scale IV.

Five patients underwent invasive hemodynamical investigations while on dobutamine, showing median mean pulmonary pressure of 25 mm Hg, median pulmonary arterial wedge pressures of 19 mm Hg, pulmonary resistances of 1 UI, and a median cardiac output of 4 L/mn (cardiac index 2 L/mn/m^2^).

Biological parameters at admission are described in Table 2.

At admission, median LVEF was 22% (IQR 11, from 10 to 35), and EDLVD 61 mm (IQR 4, from 52 to 65). Median TAPSE, S wave, and systolic pulmonary artery pressure were 14 mm (IQR 5), 9 cm/s (IQR 3.5), and 40 mm Hg (IQR 11), respectively.

Each patient fulfilled each of the FRENSHOCK registry score items. The median SAPS score was 32 (IQR 15).

All patients underwent coronary angiography as part of the etiologic work-up, without finding any significant lesion, ruling out an ischemic cardiomyopathy as the etiology of heart failure.

Nine of the 14 patients (64%) required the infusion of vasopressive agents or catecholamine support (4 dobutamine and 5 dobutamine and norepinephrine). Two patients needed a circulatory support by extracorporeal membrane oxygenation (ECMO) and invasive mechanical ventilation. One of these two patients initially experienced cardio–respiratory arrest due to electro–mechanical dissociation following intravenous amiodarone administration, which rapidly recovered after external cardiac massage and epinephrine injection. One patient experienced renal failure requiring transient renal replacement therapy with the recovery of normal renal function in a few weeks.

Prior to ablation, acute amiodarone loading and electrical cardioversion were attempted in five patients and were successful in four. Four patients received amiodarone alone, which did not convert the arrhythmia. One other patient underwent an unsuccessful external cardioversion. Beta-blockers were prescribed in five patients (35%) and worsened heart failure in all. Digoxin was used in two patients without any issue. Flecainide, sotalol or a calcium channel blocker were not used in any patient.

All 14 patients underwent catheter ablation, either during hospitalization (*n* = 8, median 2 days after admission) or shortly after discharge (*n* = 6, median 4 weeks after discharge). Patients on catecholamines or mechanical support were ablated while on these therapeutics in each case. Patients ablated in emergency during the hospitalization were those in whom ablation was considered as the last resort therapy because of failure of AA drugs or DC shocks, while patients ablated later were those in whom the initial rate or rhythm control was sufficient to improve hemodynamics enough to allow us to postpone the procedure. Despite similar LVEF at admission, LVEF at discharge was lower in patients needing urgent ablation compared to patients with ablation postponed (32 vs. 41%, *p* = 0.1). Uncomplicated ablation procedures were performed in all but one patient (false aneurysm medically managed). All ablation procedures were considered acutely successful with regain of stable sinus rhythm.

In-hospital outcomes:

The clinical status improved in all patients after resumption of sinus rhythm (or rate control for patients later ablated), allowing discharge after 10 days (IQR 9) of hospitalization (5 days in ICCU, IQR 5). At discharge, there was a significant improvement in LVEF (33% IQR 7, *p* = 0.01) and a trend in EDLVD (52 mm, IQR 7, *p* = 0.17) compared to admission.

Biologically, there were non-significant decreases in creatinine and NT-pro BNP levels, with borderline improvement in eGFR, and significant improvements in prothrombin ratio and total bilirubin (Table 2).

All patients were discharged on oral anticoagulant and beta-blockers, 13 on ACE inhibitors (severe renal failure in one patient), 5 on an aldosterone antagonist, and 12 on furosemide with a mean daily dose of 40 mg (IQR 40). Nine patients (64%) were on amiodarone at hospital discharge.

Long-term follow-up:

Two out of the 14 patients (14%) experienced recurrence of AT requiring a second ablation (10 weeks and 5 months after discharge). Of these, only one presented recurring multiple AT, needing four other ablation procedures (see flow chart Figure 1). At the end of the follow-up, all patients were in sinus rhythm.

Cardiac MRI was performed on nine patients (64%) at a median of 0 months (IQR 3) after discharge and showed a median LVEF of 37% (IQR 15) and EDLVD of 127 mL/m^2^ (IQR 42). A late gadolinium enhancement (LGE) evocative of structural cardiomyopathy was not observed in any case.

Median follow-up was 8 months (IQR 10, from 1 to 17 months). One patient moved out of the country one month after the hospitalization. No other patient was lost to follow-up.

Only one patient required hospitalizations for relapses of acute heart failure 3 and 4 months after discharge, which were concommittant to recurrences of AT who underwent new successful procedures (see above).

**Figure 2:**
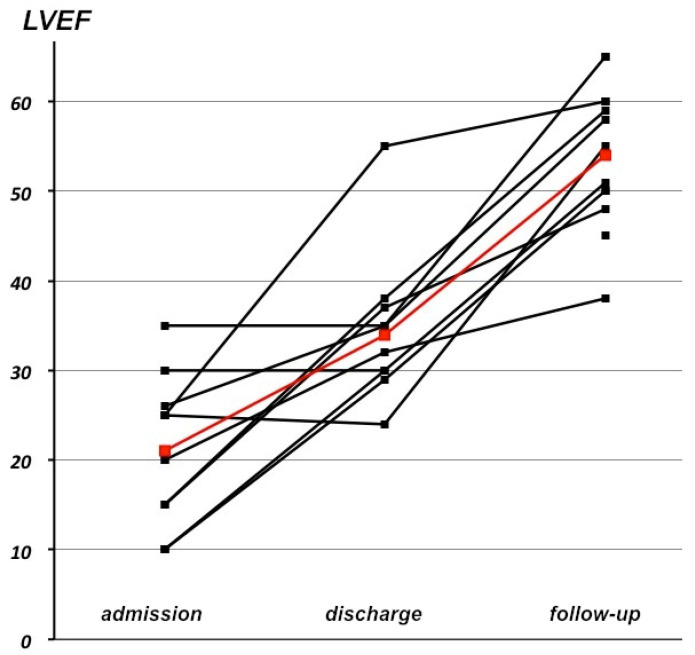
Evolution of LVEF between admission, discharge, and follow-up (red = mean).

**Figure 3:**
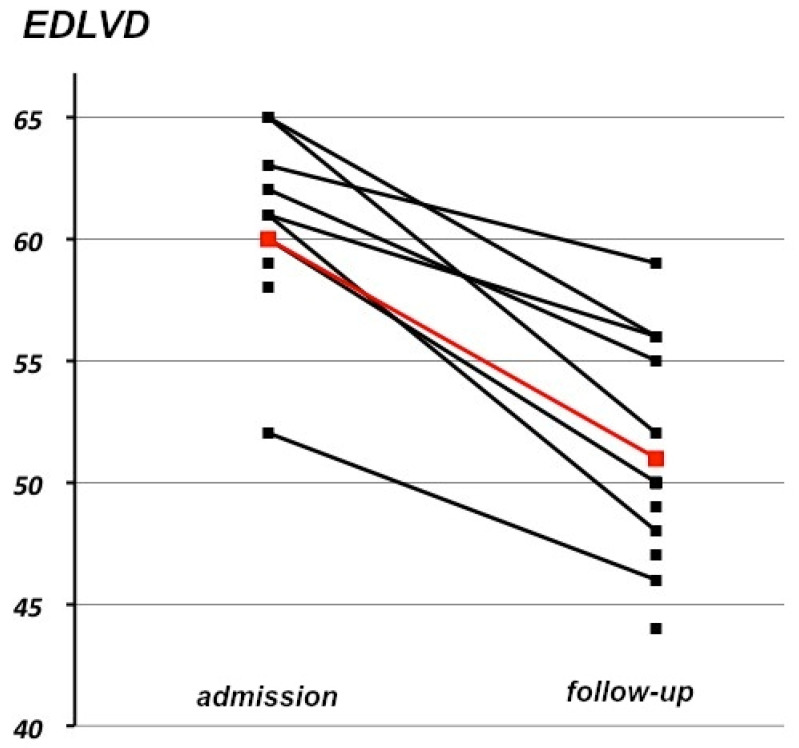
Evolution of EDLVD between admission and follow-up (red = mean).

At the end of the follow-up, the median NYHA score was 1 (IQR 1) (*p* = 0.002 compared to admission). LVEF significantly increased (55%, IQR 10) (*p* = 0.001) (Figure 2) and EDLVD significantly decreased (50 mm, IQR 8) (*p* = 0.01) (Figure 3) compared to admission. Fully normal LVEF and EDLVD were present in nine out of 13 patients.

Diuretics could be stopped in four patients due to clinical improvement. Amiodarone therapy was stopped in one patient and added in another one. Other treatments remained unchanged during the follow-up.

No patient died, and no patient experienced ventricular arrhythmia, implantation of chronic mechanical circulatory support, ICD or heart transplantation during the follow-up. There was no difference in clinical, biological, arrhythmia, and echocardiographical presentation and evolution between patients with excessive alcohol intake and others.

## 4. Discussion

In this study, we evaluated the evolution of severe TIC with cardiogenic shock because of intractable recent atrial arrhythmias (mainly AF or flutter) in a small population of 14 patients hospitalized in the Intensive Cardiac Care Unit and managed by percutaneous ablation. After one or several ablation procedures, stable sinus rhythm could be restored in all patients with major enhancement or even normalization of hemodynamical and echocardiographical conditions over a mean follow-up of 7.5 months. This strategy allowed avoiding the implantation of mechanical circulatory support (MCS) or transplantation, which had been discussed for some patients at the early phase. Even if tachycardia recurred in 15% of cases, additional procedures allowed regain of long-term stable sinus rhythm in all cases. No major complication happened due to the ablation procedure in these risky patients. The only patient requiring hospitalization for relapse of acute heart failure during the follow-up demonstrated recurrent arrhythmia and underwent new successful procedures. Thus, in this series of consecutive unselected patients with severe heart failure, high SAPS score, and recent atrial arrhythmias, the arrhythmia was clearly demonstrated to be the cause of heart failure in each case, and all patients regained normal or almost normal conditions once successfully treated.

While TIC is a well-known reversible form of heart failure and essential to diagnose in order to obtain recovery of the cardiac function, very few studies have focused on the most severe forms of TIC and therefore on the effectiveness of the available therapies (rate control or rhythm control by drugs or ablation) in this critical situation. Indeed, there are only a few reports regarding the management of TIC in the case of cardiogenic shock, and to the best of our knowledge there is no series of severe TIC in adults.

A few case reports are available in this setting. Most did not report on the effectiveness of ablation. Some related TIC complicated by cardiogenic shock in adults or children with various supraventricular tachycardia, with some needing acute MCS, with final control by drugs [14,16,20,21]. A single series is available to date, relating the efficacy of overdrive by transesophageal atrial pacing of supraventricular tachycardia in children with TIC complicated by cardiogenic shock, allowing resumption of sinus rhythm and recovery of cardiac function [22]. Cases reporting ablation for severe TIC are even more exceptional: a 64-year-old woman with cardiogenic shock secondary to permanent junctional reciprocating tachycardia successfully managed by ablation [13] and a case of intractable supraventricular tachycardia in a patient with TIC complicated by cardiogenic shock also successfully treated by ablation [23].

This study is therefore the first series of adult patients with severe TIC treated by ablation. According to our experience, we can therefore conclude that RF ablation of culprit atrial arrhythmias seems to be a reliable therapy for maintaining stable sinus rhythm while leading to clinical, hemodynamical and echocardiographical improvement or normalization of LVEF, even in the most severe cases of TIC. Catheter ablation should therefore be performed as an urgent first-line therapy, especially after failure of acute administration of anti-arrhythmic drugs, before considering acute or long-term MCS or heart transplantation in these cases. In half of the patients, ablation was not performed in emergency, but relatively early after acute improvement and discharge (a few weeks after). If recovery from cardiogenic shock was initially caused by rhythm or rate control using drugs and/or DC shock, most of the patients would have undergone relapses without early ablation in this setting of severe heart failure, where drug use is limited and the recurrence rate is high [12]. Thus ablation, either urgent or slightly delayed, was involved in short- and long-term reversal of TIC.

The important part of patients with excessive chronic alcohol intake in our series should be mentioned. Alcohol-induced cardiomyopathy is an accepted subclass of cardiomyopathy different from TIC. Further works are mandatory for investigating if excessive alcohol consumption may lead to higher sensitivity to TIC.

Worsening of hemodynamical status was observed in a third of our patients, and secondary to the introduction of anti-arrhythmic medications in each case in this context of uncontrolled heart failure. Even if they are a widely accepted therapy for heart failure, it should be recalled that beta-blockers have a negative inotropic effect and their use should be prudent in the context of acute severe heart failure [1]. Intravenous amiodarone was responsible for electro–mechanical dissociation in one of our patients, probably because of slight negative inotropic effects and/or vasodilating properties of the solvent. Previous cases also reported such adverse effect in TIC [21]. Thus, extreme caution should be used when using anti-arrhythmic drugs in severe heart failure. This is another reason to propose ablation instead of drug therapy in such severe TIC.

We did not observe ventricular arrhythmias or sudden death during the follow-up, despite initially severely altered left ventricular function and dimensions, but this complication has been described [15,24,25]. Even if regression of clinical and echocardiographical parameters were obtained by ablation, this does not mean that full normalization of structural modifications was achieved. Therefore, close prolonged follow-up of patients with TIC must be encouraged. The implantation of an ICD has been proposed in some cases, even in the case of normalization of left ventricular function and especially in the case of LGE on MRI.

Clinical improvement in TIC is usually observed within one month of treatment of the arrhythmia [26], and recovery of the left ventricular systolic function may take two to three months, but usually no more than six months [26]. Kinetics of improvement in our patients appear to be in accordance with these data. Heart failure therapies need to be maintained after ablation [26], at least until normalization of LVEF and left ventricular dimensions. In the case of relapse of arrhythmia, the alteration of LV function may occur more rapidly than during the initial episode, probably due to the persistence of myocardial abnormalities [5]. Such a case was not observed in our series, even if one patient experienced recurring heart failure due to relapses of arrhythmias.

The excellent long-term outcome of this population highlights the role of maintaining stable sinus rhythm in patients with severe TIC and especially by ablation. In non-ischemic patients, short-term prognosis is usually considered very poor after cardiogenic shock [27], and the excellent short-term outcome in this selected and ablated population highlights the crucial role of rhythm/rate control in TIC, which persisted long-term.

Differentiating TIC from idiopathic dilated cardiomyopathy would be useful for avoiding unnecessary anti-arrhythmic drug therapy or ablation, but no efficient technique is available to date to diagnose TIC “a priori”. On the other hand, maintaining stable sinus rhythm in patients with any dilated cardiomyopathy with heart failure is also of utmost importance. Furthermore, since patients with structural cardiomyopathy may also benefit from stable sinus rhythm, a certain degree of TIC is probably present in every cardiomyopathy with AF. Cardiac MRI is advisable to exclude intrinsic structural changes [28]. Although no LGE was observed in our patients, abnormal findings on MRI should not discourage one to perform rhythm control since any patient would benefit from stable sinus rhythm. The ratio of NT-proBNP at baseline versus during follow-up has also been proposed for differentiating TIC from idiopathic dilated cardiomyopathy [29]. However, there was no significant decrease in NT-proBNP in our patients; thus, this parameter does not seem to be sensitive, at least in the acute phase.

Finally, if the culprit arrhythmia cannot be ablated or if the ventricular rate cannot be controlled by drugs, it has been recommended to ablate the atrio-ventricular node and implant bi-ventricular devices [29]. However, studies and meta-analysis have shown that AF ablation provides better improvement in LVEF compared to control of the ventricular rate alone [30,31]. We did not use this technique since ablation was successful in all patients in this short series.

## 5. Limitations

This was a single center study including a limited number of patients, and these results need to be validated in multicenter prospective registries given the paucity of such severe TIC.

This study was retrospective with all the usual associated drawbacks, such as unavailable data (cardiac MRI or biology, for example) that may have altered the causal diagnosis of cardiomyopathy and its true severity. However, patients in this study were consecutive and unselected, and all of them were proved to present with TIC in view of the dramatic enhancement of symptoms and LVEF after return to stable sinus rhythm in each case.

Even if some improvement can be observed long-term using medical therapy after cardiogenic shock, such quick and major changes in LVEF, LV dimensions, and clinical status may not be solely explained by drug therapy only, but rather likely by resumption of stable sinus rhythm. Comparative studies are needed for demonstrating the true role of ablation in severe TIC, however.

Six patients presented with excessive chronic alcohol consumption. Given the similarity of clinical and echocardiographical presentations of alcohol-induced cardiomyopathy and TIC, it remains difficult to formally assess the diagnosis of TIC in these cases because interruptions of intoxication and of arrhythmia were concomitant. However, regression of alcohol-induced cardiomyopathy cannot be so rapid after cessation of intoxication, and there was no difference in any parameter and evolution compared to other patients.

## 6. Conclusions

Severe TIC with cardiogenic shock is not exceptional. Catheter ablation may be proposed early for severe refractory TIC, which allows the maintenance of stable sinus rhythm, leading to significant improvement or normalization of the left ventricular function and volumes and functional status, despite an initially expected poor prognosis.

## Figures and Tables

**Table 1:** Baseline clinical characteristics of included patients.

Body Mass Index (kg/m^2^)	27 (IQR 8)
Smokers	7 (50%)
Hypertension	4 (29%)
Diabetes	1 (7%)
Hypercholesterolemia	0
Familial ischemic heart disease	2 (14%)
Chronic obstructive pulmonary disease	1 (7%)
Excessive alcohol consumption	6 (43%)
Hypothyroidism/thyrotoxicosis	0
Coronary artery disease	none

**Table 2:** Biological characteristics of included patients and evolution from admission to discharge (results in median and IQR).

	at Admission	at Discharge	*p*
pH	7.39 (IQR 0.1)		
TSH (mUI/L)	3.8 (IQR 2.8)		
Lactate (mmol/L)	5 (IQR 5.6)		
Creatinine (µmol/L)	116 (IQR 47)	88 (IQR 24)	0.14
Estimated glomerular filtration rate (mL/min/m^2^)	58 (IQR 27)	82 (IQR 37)	0.07
NT-proBNP (pg/mL)	3325 (IQR 3725)	990 (IQR 625)	0.16
ASAT (UI/L)	65 (IQR 2158)		
ALAT (UI/L)	65 (IQR 1094)		
Factor V Leiden concentration (%)	34 (IQR 45)	107 (IQR 48)	ns
Total bilirubin (mmoL/L)	23 (IQR 27)	11 (IQR 8)	0.002
Prothrombin ratio (%)	58 (IQR 40)	83 (IQR 30)	0.005

## Data Availability

Data available on request.
